# Machine learning-based delirium prediction in surgical in-patients: a prospective validation study

**DOI:** 10.1093/jamiaopen/ooae091

**Published:** 2024-09-17

**Authors:** Stefanie Jauk, Diether Kramer, Stefan Sumerauer, Sai Pavan Kumar Veeranki, Michael Schrempf, Paul Puchwein

**Affiliations:** Division of Technology and IT, Steiermärkische Krankenanstaltengesellschaft m.b.H. (KAGes), 8010 Graz, Austria; PH Predicting Health GmbH, 8010 Graz, Austria; Division of Technology and IT, Steiermärkische Krankenanstaltengesellschaft m.b.H. (KAGes), 8010 Graz, Austria; PH Predicting Health GmbH, 8010 Graz, Austria; Department of Neurology, Steiermärkische Krankenanstaltengesellschaft m.b.H. (KAGes), 8036 Graz, Austria; Division of Technology and IT, Steiermärkische Krankenanstaltengesellschaft m.b.H. (KAGes), 8010 Graz, Austria; PH Predicting Health GmbH, 8010 Graz, Austria; PH Predicting Health GmbH, 8010 Graz, Austria; Department of Orthopaedics and Trauma, Medical University of Graz, 8036 Graz, Austria

**Keywords:** random forest, prediction algorithms, electronic health records, clinical decision support, delirium

## Abstract

**Objective:**

Delirium is a syndrome that leads to severe complications in hospitalized patients, but is considered preventable in many cases. One of the biggest challenges is to identify patients at risk in a hectic clinical routine, as most screening tools cause additional workload. The aim of this study was to validate a machine learning (ML)-based delirium prediction tool on surgical in-patients undergoing a systematic assessment of delirium.

**Materials and Methods:**

738 in-patients of a vascular surgery, a trauma surgery and an orthopedic surgery department were screened for delirium using the DOS scale twice a day over their hospital stay. Concurrently, delirium risk was predicted by the ML algorithm in real-time for all patients at admission and evening of admission. The prediction was performed automatically based on existing EHR data and without any additional documentation needed.

**Results:**

103 patients (14.0%) were screened positive for delirium using the DOS scale. Out of them, 85 (82.5%) were correctly identified by the ML algorithm. Specificity was slightly lower, detecting 463 (72.9%) out of 635 patients without delirium. The AUROC of the algorithm was 0.883 (95% CI, 0.8523-0.9147).

**Discussion:**

In this prospective validation study, the implemented machine-learning algorithm was able to detect patients with delirium in surgical departments with high discriminative performance.

**Conclusion:**

In future, this tool or similar decision support systems may help to replace time-intensive screening tools and enable efficient prevention of delirium.

## Background and objectives

Surgical treatment of mentally vulnerable patients like elderly people, addicts, or people with mental disorders is becoming increasingly demanding due to multi-morbidity and polypharmacy of those patients. Delirium is one of the major complications after surgery, especially in hip fractures.^1^^,^^2^ The syndrome can be expected in 13%-70% of older patients with hip fractures and is associated with poor outcome and an increased mortality.^3^ Demographic changes in countries around the world will raise the number of patients in risk dramatically. Therefore early detection of patients in risk and proactive measures to prevent delirium is mandatory.

Although a single factor can contribute to delirium, its onset is commonly multifactorial.^4^ The multiplicity of reasons and cofounding factors makes predicting delirium a real challenge in acute care settings, and thus delirium is frequently under-recognized.^5^^,^^6^ Despite the fact that delirium-screening tools can increase the detection rate, the screening is often inconsistent. Particularly in surgical wards, the screening and recognition of delirium is limited.^7–9^ The screening rate for delirium in surgical wards is between 10% and 30%, whereas 77%-100% stay unrecognized.^10^ This may be related to the facts that the optimal care of patients with a higher risk of delirium is a resource-intensive process and the time of health care staff is a limited resource.

Delirium is a syndrome that leads to severe complications for the person affected. Since it is considered preventable in many cases, a well-founded prevention program could improve health care quality in many aspects. In fact, there are also numerous pharmacological as well as nonpharmacological preventive measures.^4^^,^^11^ In the context of scarce resources these measures must be used in a targeted manner to be deployed optimally. Thus, one of the biggest challenges is to identify the patient at risk in a hectic clinical routine. Existing screening tools like the DOS scale^12^ and the confusion assessment method (CAM)^13^ are time consuming and are rather used for assessing first signs and symptoms of delirium than for prediction.

### The use of machine learning for the prediction of delirium

With the increasing amount of clinical data stored in electronic health record (EHR) systems, machine learning (ML)-based prediction models have become popular for the prediction of clinical outcomes.^14^^,^^15^ Compared to screening scales, ML models are less limited with respect to accuracy, since most of these methods are able to learn complex relationships. Although many well performing ML models have been published over the last years, few models have been deployed in clinical routine.^16^^,^^17^ In order to assess the actual potential of ML-based prediction in clinical settings, there is an urgent need for prospective validation studies.^18^

Over the last years, various prediction models for delirium have been developed, but had flaws regarding their reliability for clinical use,^19^^,^^20^ or generalizability for other populations.^21^ The model from Wong and colleagues,^22^ a Gradient Boosting Machine model, achieved an Area Under the Receiver-Operating Characteristic (AUROC) of 0.86 on a test dataset when predicting delirium for non-ICU patients at hospital admission. A random forest model of Corradi and colleagues^23^ predicted a positive CAM assessment 48 h after admission with an AUROC of 0.861 on test data.

Although the best performing ML models predicting delirium based on EHR data achieved AUROC values up to 0.94 in test data sets, few models were subject to external validation or underwent prospective evaluation in clinical settings.^24^ Pagali and colleagues^25^ prospectively validated a prediction tool for delirium risk which included models for medical and surgical hospitalized older patients. A modified version of their tool achieved an AUROC of 0.80, which increased to 0.82 after recalibration in a validation cohort of 8055 patients. Sun and colleagues^26^ implemented ML models for several clinical outcomes in three hospitals in Germany. Their model predicting delirium at the evening of admission achieved an AUROC of 0.809 when tested on prospective clinical data. Fliegenschmidt and colleagues^27^ evaluated their models predictive performance for postoperative delirium on retrospectively gathered dataset for 114 cardiac surgery patients from a database for anesthesia quality assurance and achieved a pre-operative prediction AUROC between 0.55 and 0.66 and a post-operative prediction scenario an AUROC of 0.79.

We recently published an evaluation study of a prediction tool using random forest models and EHR data for identifying patients at risk of delirium during hospitalization.^28^ The delirium risk is predicted automatically at admission using routinely documented data such as demographics, ICD-10 coded diagnoses, laboratory data, nursing assessment, procedures, and medication. During 7 months of prospective evaluation in internal and surgical departments, the algorithm demonstrated very high predictive performance with an AUROC of 0.86, a sensitivity of 74.1% and a specificity of 82.2%.

A major limitation of our last evaluation study^28^ was a low incidence of delirium with 1.5% in the study cohort. Patients with delirium were identified using ICD-10 coded diagnoses and indication of delirium in clinical texts. However, many cases of delirium are not being detected in clinical routine,^29^ and delirium is thus often under-recorded in EHR system.

To overcome this limitation, the aim of this study was to evaluate the delirium prediction tool in clinical routine during a structured and ongoing delirium assessment using state of the art methods. The DOS scale was used to identify patients with delirium in order to validate the predictions of the ML-based tool in a real-world setting.

## Research design and methods

### Study design

The observational cohort study was conducted over 3 months at the University Hospital Graz, Austria. It received approval from the Ethics Committee of the Medical University of Graz (30-146 ex 17/18). Three departments were included in the study: the vascular surgery department, the orthopedic surgery department, and the trauma surgery department. Altogether, the three departments treat around 5000 in-patients every year.

All patients admitted to one of the surgical departments between November 2020 until January 2021 were included in the study; this included both acute and elective patients. Patients below the age of 18 were excluded from the study.

For all patients, an automated prediction was performed by the delirium prediction tool. Although the tool had been in use before November 2020 at the surgical departments and the results had been displayed in the hospital information system (HIS), the prediction was conducted in the background during the validation study. The prediction results were not visible to health care professionals and the DOS assessment was conducted without influence of the ML prediction.

### Assessment of delirium

The discriminative performance of the tool was evaluated on the results of the DOS scale used for delirium screening. The DOS scale has been designed to allow a fast and easy identification of delirium and is based on the DSM-IV criteria for delirium.^12^ It has a high sensitivity and specificity for predicting delirium in hospitalized patients,^30^ and has demonstrated high interrater reliability agreement among registered nurses in an Austrian study.^31^ Nurses participating in this study had been using the DOS scale in previous studies and were familiar with it.

A DOS sum equal or greater than 3 points was defined as an occurrence of delirium. For each patient admitted to one of the departments, the DOS scale was conducted twice a day from the day of admission until 3 days after surgery. In case of a second surgery, the screening was conducted again from the day of surgery until 3 days after. This restriction was made due to limited resources in the departments; however, in case of any symptoms of delirium, the DOS assessment was continued or restarted for the patients. Printed questionnaires were distributed at the departments and completed by nursing staff, who was trained on the DOS scale beforehand. Besides the DOS scale, the nurses documented hypoactive behavior of the patients and the use of any sedatives.

In order to compare the results of the DOS assessment, EHR data were screened for any evidence of delirium. ICD-10 coded delirium diagnoses were extracted from the EHR system and free-text patient summaries including discharge summaries and psychiatric consultation reports were searched for indication of delirium.

### Delirium prediction tool

The Personalised Risk Tool is a software predicting the individual risk of patients in hospitals, nursing homes, and private practices in order to better target available resources. The software calculates a patient’s risk for the occurrence of a disease, a complication or adverse clinical event such as delirium, dysphagia, or intensive care unit admission. The Personalised Risk Tool has been first deployed in hospitals of the public health care provider Steiermärkische Krankenanstaltengesellschaft m.b.H. (KAGes) in the province of Styria, Austria, in 2018, and has been expanded to various other hospitals across Austria since then.

The random forest models integrated in the tool had been trained on data of more than 19 000 patients admitted to different departments of KAGes hospitals between January 2011 and March 2019. Due to the coverage of over 90% of all hospital beds in Styria, KAGes has access to more than 2 million longitudinal patient histories. The models used routinely documented data available in the EHR system including demographic data (eg, age, sex), transfer data (eg, ward of admission), ICD-10 coded diagnoses, laboratory data (mapped to international LOINC, logical observation identifiers names and codes) procedures (mapped to Austrian procedure codes), nursing assessment (eg, visual impairment), and prescribed drugs (mapped to ATC, Anatomical therapeutic chemical, Classification).

The models were trained with the R package *randomForest* included in the *caret* package^32^ using an up-sampling method, a 10-fold cross-validation and a 75/25 train-test-split. The binary outcome was delirium documented during a hospital stay. This included (1) ICD-10 coded delirium (code F05.x) and alcohol withdrawal state with delirium (code F10.4), and (2) delirium reported in clinical notes (eg, discharge summaries, psychiatric notes). The most important features of the models were identified using the *varImp* function from the *caret* package for random forest models (see Figure SA1).

To provide a high clinical utility, the thresholds for risk stratification were determined in discussion with health care professionals. Cut-offs for the risk groups were set based on the risk probabilities predicted on a sub-dataset of the participating clinical departments at the 85th and 95th percentile. The delirium prediction by the software was performed automatically for every patient admitted to any of the 3 surgical departments; an HL7 transaction was sent from the HIS to a local hospital server, and patient data needed for prediction was retrieved from the EHR system using http-requests. The algorithm predicted delirium for each patient at (1) admission time, (2) the evening of admission, and (3) the second evening, including the most recent laboratory results and nursing assessment data. All risk predictions and features values were stored in a database.

### Data analysis

The data was analyzed in *R* Version 3.6.2. Descriptive statistics of the included patients were extracted from the EHR system of the hospital network. In order to identify previous diagnoses of the patients, relevant ICD-10 codes were retrieved.

Completed DOS questionnaires were digitalized in a tabular format for data analyses with 1 data entry for each DOS assessment. Hence, various DOS assessments per patient were present in the dataset. For the comparison with the prediction algorithm, any positive result of the DOS scale of a patient’s hospital stay was defined as case of delirium.

As the admission time varies between the patients, only the latest prediction within the first 48 hours of the hospital stay was used for comparison with the DOS scale. For all patients, this corresponded to the prediction on the second evening of the hospital stay. As the algorithm stratifies patients into 3 risk groups, the “high risk” and “very high risk” group needed to be combined and defined as delirium positive. Based on these 2 groups, sensitivity, specificity, positive predictive value, negative predictive value, and accuracy were calculated for the prediction algorithm.

As a measure of discrimination, ROC curves with DeLong confidence intervals^33^ were used. ROC curves present the sensitivity and specificity at varying threshold values. In addition, a calibration plot with a 95% confidence interval was computed illustrating the frequencies of delirium cases over the probabilities predicted by the algorithm.^34^ In a calibration plot, the estimated risk (on the *x*-axis) is compared to the observed proportion of events (*y*-axis); a curve on the diagonal would illustrate excellent calibration. Furthermore, the brier score^35^ was reported as an overall assessment of model calibration and discrimination, with a lower score indicating a better prediction result. The brier score was calculated using the *DescTools* package, and the scaled brier score using the *psfmi* package^36^ in *R*.

## Results

During the 3-months study period, 738 patients were admitted to one of the participating departments. Although the median age was highest for the vascular surgery department with 71 years, the third quartile was highest for the trauma surgery patients with 25% older than 81 years (Table 1).

**Table 1.** Descriptive statistics of the patients included at three surgical departments (*n* = 738).

		Vascular surgery		Orthopedic surgery		Trauma surgery		Total	
*n*		214		243		281		738	

		*n*	%	*n*	%	*n*	%	*n*	%
Sex	m	139	65.0	118	48.6	145	51.6	402	54.5
	f	75	35.0	125	51.4	136	48.4	336	45.5

		***Median***	***(Q1-Q3)***	***Median***	***(Q1-Q3)***	***Median***	***(Q1-Q3)***	***Median***	***(Q1-Q3)***

Age (years)		71	(62-76)	61	(50-73)	68	(50-82)	67	(53-77)
BMI		26	(23-29)	27	(24-31)	25	(22-28)	26	(23-29)
Longest hospital stay (days)^a^		9	(4-15)	6	(3-14)	8	(4-14)	8	(3-14)

		***Mean***	***(Min, max)***	***Mean***	***(Min, max)***	***Mean***	***(Min, max)***	***Mean***	***(Min, max)***

Number of admissions^a^		2,55	(0,13)	1,80	(0, 9)	1,97	(0, 22)	2,08	(0, 22)
Number of ICD-coded diagnoses^a^		10,27	(0, 47)	7,73	(0, 42)	7,00	(0, 45)	8,30	(0, 47)
Charlson Comorbidity Index		4,61	(0, 25)	2,44	(0, 21)	2,67	(0, 20)	3,35	(0, 25)

a Within the last 3 years.

Overall, 103 patients (14.0%) had a DOS sum higher or equal to 3 within their hospital stay and where thus identified as patients with delirium (Table 2). The trauma surgery department had the highest incidence of delirium with 22.8%. ICD-10 coded delirium diagnoses or indication of delirium in discharge letters were only documented for 21 patients, and 26 patients had a psychiatric consultation. Hypoactiveness was documented for 48 patients (6.5%), and sedatives were administered to 33 patients (4.5%), with the most common ones being quetiapine, risperidone, and lorazepam. For patients older than 80 years, incidence of delirium was higher than 18% for all 3 departments (see Table SA1).

**Table 2.** Incidence of delirium according to the DOS assessment and routinely documented information from the EHR system.

		Vascular surgery		Orthopedic surgery		Trauma surgery		Total	
*n*		214		243		281		738	

		*n*	%	*n*	%	*n*	%	*n*	%
DOS assessment	No delirium	187	87.4	231	95.1	217	77.2	635	86.0
	Delirium	27	12.6	12	4.9	64	22.8	103	14.0

Discharge summary/ICD-10 coded^a^	No delirium	217	100.0	236	97.1	267	95.0	717	97.2
	Delirium	0	0.0	7	2.9	14	5.0	21	2.9

Psychiatric consultation^a^	No delirium	212	99.1	238	97.9	262	93.2	712	96.5
	Delirium	2	0.9	5	2.1	19	6.8	26	3.5

a Information retrieved from EHR system after discharge.

**Figure 1.**
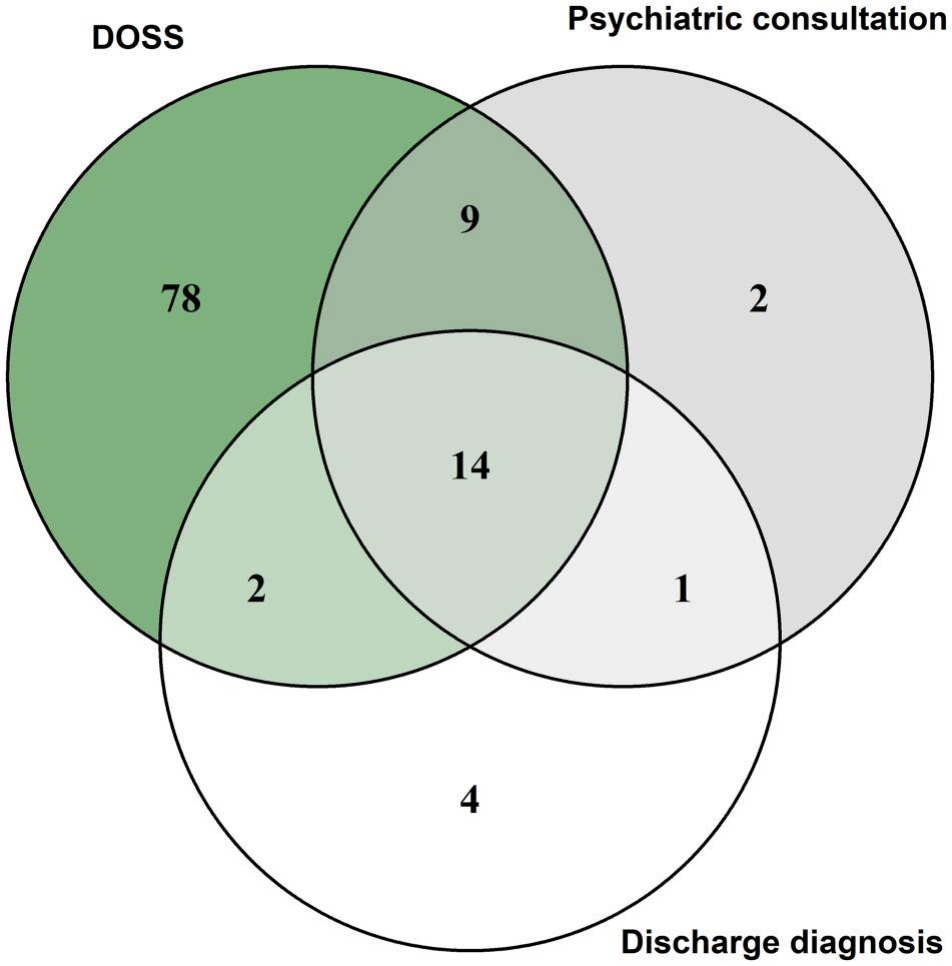
Agreement between DOS results and EHR documentation for 110 patients. 103 patients were rated as DOS positive, 7 patients had a documentation of delirium in the EHR system (psychiatric consultation indicating delirium or discharge diagnosis for delirium).

Out of all 103 patients with delirium according to DOS, 16 had a discharge diagnosis for delirium (ICD-10 coded or in discharge letter), and 23 had a psychiatric consultation with documentation of delirium (see Figure 1). Seven patients of the study cohort had a negative DOS result, but documentation of delirium in the EHR (psychiatric consultation and/or discharge diagnosis). Patients 1-5 had long hospital stays and the delirium occurred more than 3 weeks after the DOS screening. While Patient 1 had a cefepime-induced delirium, Patient 2 and Patient 5 had COVID-19 during the episode of delirium. Patients 3 and 5 died during the hospital stay. Patient 6 showed first signs of delirium four days after surgery, when no further DOS assessment was conducted. Patient 7 had a short episode of delirium during 1 night and was administered lorazepam. However, the documented DOS sum during the night of delirium was only 1 point.

The algorithm predicted delirium with high risk for Patient 2, 3, and 6 and a very high risk for Patient 4 and 5. For Patient 1 with cefepime-induced delirium and Patient 7 with a short episode of delirium, no delirium risk was predicted by the algorithm.

### Prospective performance of the delirium algorithm

**Figure 2.**
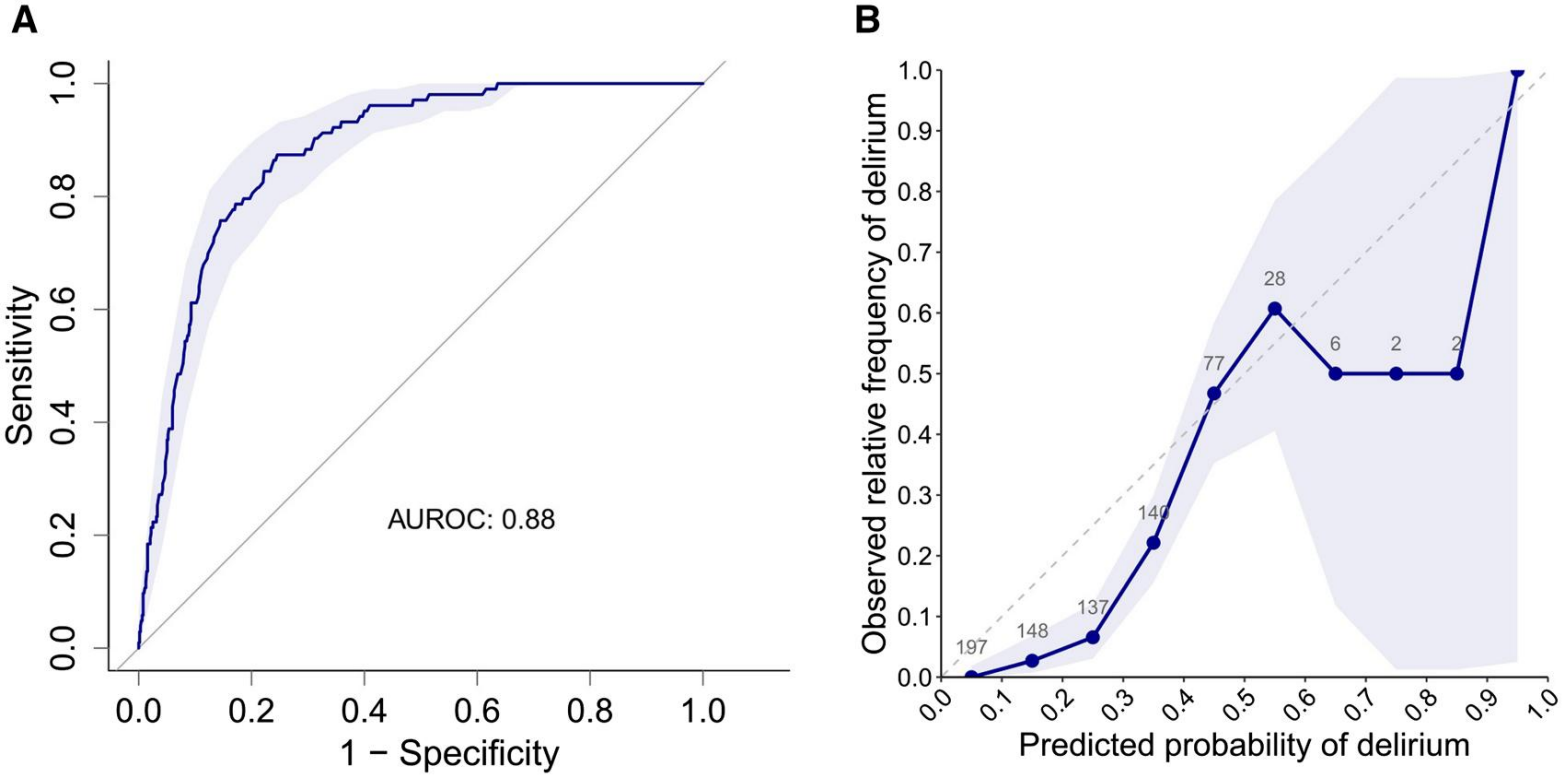
(A) Discriminative performance illustrated with an ROC curve and (B) calibration plot for all patients included in the study (*n* = 738).

During the prediction in clinical routine, the algorithm achieved a sensitivity of 82.5% and a specificity of 72.9% (see Table 3). The positive predictive value was 0.331, the negative predictive value was 0.963 and the accuracy was 0.743. Figure 2 shows the ROC curve (a) and the calibration plot (b) for the study cohort. ROC curves and calibration plots for each of the 3 departments are included in Figure SA2. The AUROC was 0.883 [95% CI: 0.8523-0.9147] for all surgical patients; AUROC was 0.797 [95% CI: 0.7040-0.8894] for vascular surgery patients, 0.898 [95% CI: 0.8247-0.9722] for orthopedic surgery patients and 0.873 [95% CI, 0.8319-0.9140] for trauma surgery patients.

**Table 3.** Confusion matrix comparing the prediction of the ML algorithm with the occurrence of delirium assessed by the DOS scale. The specificity of 72.9% and the sensitivity of 82.5% are highlighted in bold.

		Algorithm prediction					
		**No delirium** (Low risk)		**Delirium** (High/very high risk)		Total	
		*n*	%	*n*	%	*n*	%
DOS scale	No delirium	463	**72.9**	172	27.1	635	100.0
	Delirium	18	17.5	85	**82.5**	103	100.0
	Total	481	65.2	257	34.8	738	100.0

Values are presented as absolute frequencies and row percentages.

The brier scores (scaled brier scores) were 0.105 (0.126) for the entire study cohort, 0.118 (−0.071) for vascular surgery patients, 0.054 (−0.160) for orthopedic surgery patients and 0.139 (0.212) for trauma surgery patients.

## Discussion and implications

The results of this study demonstrate the high discriminative performance of a ML-based delirium prediction tool when used for surgical in-patients. Over a 3-months period, automated real-time predictions were compared to a systematic delirium assessment using the DOS scale for 738 patients of 3 surgical departments, demonstrating a sensitivity of 82.5% and a specificity of 72.9%. While the DOS assessment, which was performed twice a day over several days of the hospital stay, created an additional workload for health care professionals, the ML-based prediction was performed automatically without any additional effort in a few seconds after admission.

Many delirium models published use strict inclusion criteria such as older adults, patients without dementia or previous episodes of delirium or patients from a certain field only. However, the random forest models used in this study were trained on a heterogeneous dataset of over 19 000 in-patients from different specializations. Our goal was to develop prediction models which are able to predict delirium across different patient populations. This facilitates the deployment in clinical settings and increases the usability in clinical workflows, as there are no restrictions for predictions within one ward (eg, age restriction). In this validation study, the tool demonstrated an excellent performance for vascular and trauma surgery patients, and an outstanding performance for orthopedic surgery patients (AUROC = 0.90). Although there is evidence that the use of heterogeneous training data reduces biases of ML models,^37^ further studies should address potential biases in the implemented models as they were trained on data from one geographical region only. Few research studies demonstrate the clinical validation of ML-based delirium prediction. The model of Pagali and colleagues^25^ achieved an AUROC of 0.82 in a validation cohort of 8055 patients, and Sun and colleagues^26^ reported an AUROC of 0.81 when tested on prospective clinical data. The discriminative performance of our prediction tool in this study was higher with an AUROC ranging from 0.80 to 0.90 for the surgical departments.

### Strengths and weaknesses

A main strength of this study is the validation of a delirium prediction algorithm in a clinical setting. The majority of ML applications in this field have not been implemented nor been using real-time data for evaluation.^24^ This may lead to biased results, for example, if EHR data is not available at the point in time for prediction during clinical routine.^38^ In this study, all predictions were performed in real-time using already available EHR data for prediction. Delirium risk was first predicted at time of admission or transfer to the surgical department and re-calculated twice; on the first evening and on the second evening. These re-calculations ensured that latest information such as nursing assessment and laboratory data were included in the prediction. Another major strength is the systematic assessment of delirium over a period of three months for all patients of the surgical departments. In our first evaluation study, we compared the risk prediction of the ML algorithm with records of delirium in the EHR system. However, this leads to biased results: Delirium in hospitalized patients is often undetected or under-diagnosed,^29^ and, thus, EHR data might be incomplete. In this study, the systematic DOS screening across all in-patients demonstrated an incidence of delirium between 5% and 23%. These results are in line with recent studies on the occurrence of delirium in patients aged 65 years and older with incidences of post-operative delirium of 22% for emergency orthopedic surgery, 18% for elective orthopedic surgery and 14% for vascular surgery.^39^

A third advantage of this study was the access to the EHR data of all patients included in the study which enabled retrospective analyses on top of the prospective DOS assessment. For seven patients of the study cohort, DOS results were negative but delirium was documented in the EHR system as discharge diagnosis or within a psychiatric consultation. The delirium algorithm correctly identified 5 out of them as patients with delirium risk at time of admission, but they were overseen in the DOS screening. The 2 patients who were neither detected by DOS assessment nor by the algorithm had (1) a short episode of delirium (symptoms for only one night) and (2) a cefepime-induced delirium. As the delirium algorithm predicts already at the beginning of a hospital stay and does not account for administered medication during the stay, such cases are hard to detect.

There are several limitations to this study. First, the Personalised Risk Tool predicts the risk of delirium for the current hospital stay, but many precipitating risk factors^4^ or environmental risk factors^40^ can trigger an onset of delirium. Using EHR data, it is difficult to account for environmental risk factors such as family visits, absence of clock or reading classes, but also for precipitating risk factors such as infection and use of urinary catheter, especially when predicting at time of admission. Hence, some patients might have predisposing risk factors for delirium, but, fortunately, the triggering event during the hospital stay was missing. In this study, we could not account for such cases, as we did not systematically evaluate all false positive cases and their predisposing factors of delirium.

This leads to the second shortcoming of this study, the assessment of delirium using the DOS scale. Although the DOS scale achieves high accuracy for detecting delirium in several studies.^30^^,^^41^ DOS screening is not equivalent to a psychiatric delirium diagnosis. Nursing staff was trained in applying the DOS scale, but due to staff changes or short resources the assessment quality might vary. Furthermore, some cases of delirium might also have been missed in the study, as the systematic assessment continued for only three days post-surgery.

Third, the risk of delirium can be substantially reduced using non-pharmacological actions.^11^ Such actions were not systematically documented for in-patients included but could have affected the results of this study. Although we were aware of this before designing the study, it was not feasible for this study to document all undertaken preventive actions. In contrast to pharmacological actions, usually very common psychotropic drugs, which are recorded in the EHR system, nonpharmacological actions for delirium can be very broad and depend not only on the patients treated but also on the resources available in the hospital department. Thus, an excessive documentation of all preventive actions was beyond the scope of this study.

### Future research opportunities

Based on our study, several research questions should be addressed in future. First, external validation of the tool can inform about how generalizable the delirium predictions are for other patient groups, hospitals, or even in other countries. Although the Personalised Risk Tool showed a high discrimination in an external validation in Austria,^42^ further studies need to be conducted in countries with different cultural backgrounds and health care systems.

Besides an external validation in other countries, an opportunity for future developments is the use of federated learning (FL). FL supports model training with data from various partners maintaining privacy.^43^^,^^44^ Instead of sharing data, models are trained at each partner location and model parameters are shared. In the future, such methods will help to develop more generalizable risk prediction models, thus making it easier to distribute successful decision support tools among hospital providers.

Second, the use of ML for healthcare applications requires explainable predictions. Healthcare professionals need to validate the results of the models and identify the factors influencing a model’s prediction. Various explainable AI (XAI) techniques have been proposed using global feature importance such as SHAP values^45^ or local feature importance such as LIME.^46^ Further research needs to be conducted applying XAI methods to the ML models validated in this study.

Third, future studies should assess how the validated models influence the care processes in the departments and the impact on the predicted outcome. Evaluating the clinical impact of delirium prediction models is challenging due to several factors. There is a variability in screening and diagnosing delirium and no standardized process in clinical practice. Delirium has a multifactorial etiology including predisposing and precipitating risk factors,^4^ and thus requires multicomponent approaches for prevention and treatment. Furthermore, delirium affects different patient populations and a clinical evaluation needs to account for different subtypes of delirium.^47^ Due to this complexity, assessing the true clinical impact of our tool will require a comprehensive outcome tracking and an extensive data collection including preventive actions and environmental factors in the hospitals.

## Conclusion

This study demonstrates the high sensitivity and specificity of a ML-based delirium prediction tool during a validation at three surgical departments. During the first hours of admission, the tool was able to detect 82.5% of patients who developed delirium as defined by the DOS scale during their hospital stay. The major strength of this study was the systematic assessment of delirium for all patients admitted to the departments in order to validate the predictions of the tool. The delirium prediction was performed automatically, and without any additional effort by healthcare professionals. Hence, in future this tool or similar decision support systems may help to ensure patient safety despite the growing population of older adults and shortcomings of hospital resources.

## Supplementary Material

ooae091_Supplementary_Data

## Data Availability

The data that support the findings of this study are available from KAGes (Steiermärkische Krankenanstaltengesellschaft m.b.H., Stiftingtalstraße 4, 8010 Graz, Austria) but restrictions apply to the availability of these data, which were used under license for the current study, and so are not publicly available. Data are however available from the authors upon reasonable research proposals and with permission of KAGes.
