# Supplementary material for: Machine learning-based delirium prediction in surgical in-patients: a prospective validation study
Source: JAMIA Open. 2024 Sep 17;7(3):ooae091. doi: 10.1093/jamiaopen/ooae091 (PMC11408728; doi:10.1093/jamiaopen/ooae091)

**Supplementary File**

Supplementary Table A1. Results of delirium screening with DOS scale for patients of the three surgical departments stratified by age groups.

|  |  | **Vascular Surgery** | | | |  | **Orthopaedic surgery** | | | |  | **Trauma Surgery** | | | |
| --- | --- | --- | --- | --- | --- | --- | --- | --- | --- | --- | --- | --- | --- | --- | --- |
| **Delirium (DOS)** | | **No** | | **Yes** | |  | **No** | | **Yes** | |  | **No** | | **Yes** | |
|  |  | ***n*** | **%** | ***n*** | **%** |  | ***n*** | **%** | ***n*** | **%** |  | ***n*** | **%** | ***n*** | **%** |
| **Age  group** | <= 50 | 17 | 100.0 | 0 | 0.0 |  | 62 | 100.0 | 0 | 0.0 |  | 76 | 100.0 | 0 | 0.0 |
|  | 51 - 60 | 26 | 96.3 | 1 | 3.7 |  | 57 | 100.0 | 0 | 0.0 |  | 43 | 95.6 | 2 | 4.4 |
|  | 61 - 70 | 52 | 91.2 | 5 | 8.8 |  | 47 | 95.9 | 2 | 4.1 |  | 28 | 90.3 | 3 | 9.7 |
|  | 71 - 80 | 76 | 86.4 | 12 | 13.6 |  | 50 | 90.9 | 5 | 9.1 |  | 38 | 70.4 | 16 | 29.6 |
|  | 81 - 90 | 15 | 65.2 | 8 | 34.8 |  | 13 | 81.3 | 3 | 18.8 |  | 25 | 44.6 | 31 | 55.4 |
|  | 91 - 100 | 1 | 50.0 | 1 | 50.0 |  | 2 | 50.0 | 2 | 50.0 |  | 7 | 36.8 | 12 | 63.2 |
| **Total** |  | **187** | **87.4** | **27** | **12.6** |  | **231** | **95.1** | **12** | **4.9** |  | **217** | **77.2** | **64** | **22.8** |

**Supplementary Fig. A1.** Feature importance plot illustrating the 30 most important predictors in the random forest model predicting delirium coded with F05.


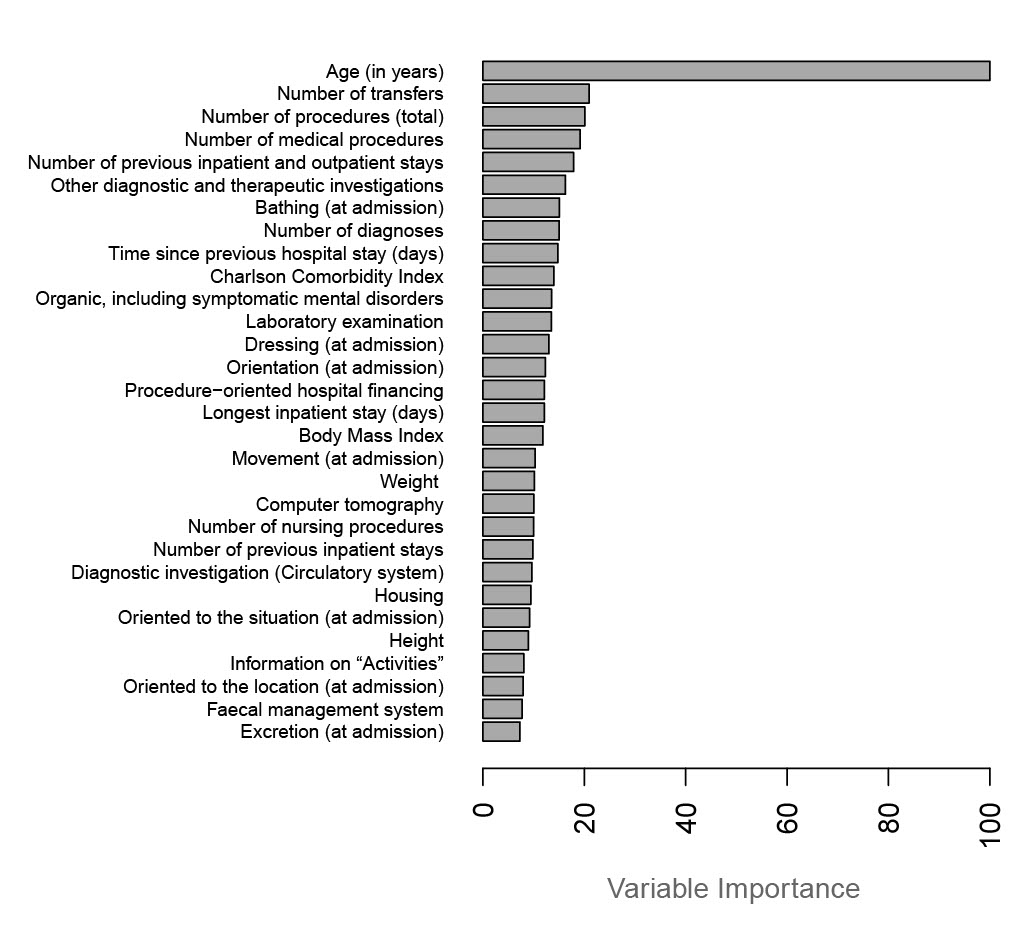


**Supplementary Fig. A2.** ROC curves and calibration plots for patients of the vascular surgery department (a-b), of the orthopaedic surgery department (c-d) and the trauma surgery department (e-f).


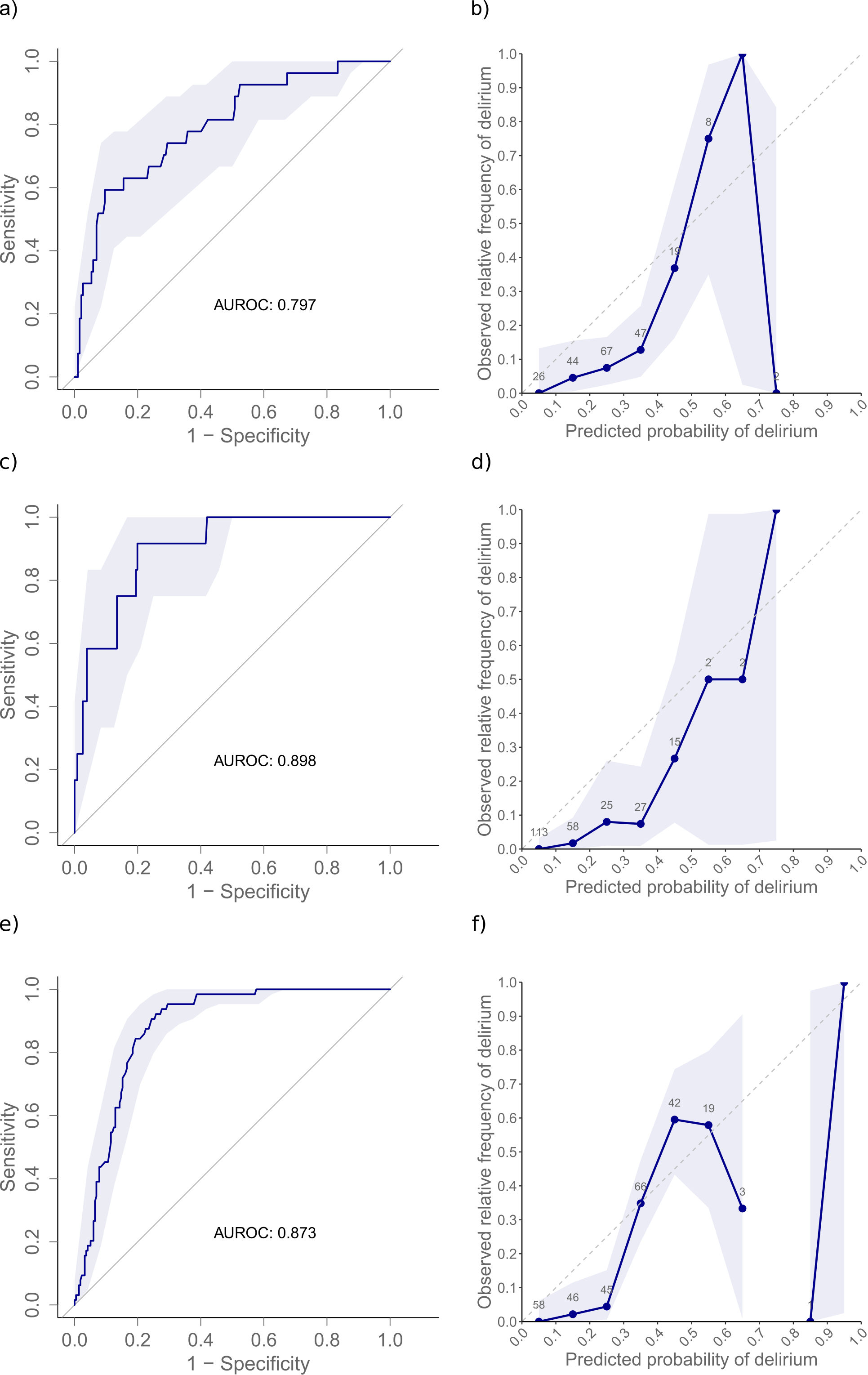

Supplement: ooae091_Supplementary_Data [file ooae091_supplementary_data.docx]
